# Evaluation of an Adaptive Implementation Program for Cognitive Adaptation Training for People With Severe Mental Illness: Protocol for a Randomized Controlled Trial

**DOI:** 10.2196/17412

**Published:** 2020-08-24

**Authors:** Michelle Thalia van Dam, Jaap van Weeghel, Stynke Castelein, Gerdina Hendrika Maria Pijnenborg, Lisette van der Meer

**Affiliations:** 1 Department of Rehabilitation Lentis Psychiatric Institute Zuidlaren Netherlands; 2 Rob Giel Research Center, University Medical Center Groningen University of Groningen Groningen Netherlands; 3 Parnassia Group Parnassia Noord-Holland Castricum Netherlands; 4 Department of Social and Behavioural Sciences Tranzo Scientific Center for Care and Wellbeing Tilburg University Tilburg Netherlands; 5 Research Department Lentis Psychiatric Institute Groningen Netherlands; 6 Department of Clinical Psychology and Experimental Psychopathology Faculty of Behavioural and Social Sciences University of Groningen Groningen Netherlands; 7 Department of Clinical and Developmental Neuropsychology Faculty of Behavioural and Social Sciences University of Groningen Groningen Netherlands; 8 Department of Psychotic Disorders GGZ Drenthe Assen Netherlands

**Keywords:** implementation, dissemination, process evaluation, severe mental illness, cognition

## Abstract

**Background:**

Cognitive Adaptation Training is a psychosocial intervention that focuses on reducing the negative effects of cognitive disorders, especially executive functions such as planning and targeted action. International research has shown that Cognitive Adaptation Training enhances multiple aspects of daily functioning in people with severe mental illnesses. Despite this evidence, implementation of the intervention into routine care remains a challenge.

**Objective:**

In this implementation research, a newly developed implementation program based on previous experience and scientific literature, is tested. The primary aim of this research is to assess the effectiveness of the implementation program. The secondary aim of this study is to evaluate the factors that impede or facilitate the implementation of Cognitive Adaptation Training.

**Methods:**

To test the effectiveness of the implementation program, a multicenter cluster randomized controlled trial was conducted comparing the implementation program to a single training program in four mental health institutions in The Netherlands. Focus groups, semistructured interviews, and questionnaires were used at multiple levels of service delivery (service user, professional, team, organization) to identify factors that may hamper or facilitate implementation. The RE-AIM framework was applied to measure the implementation effectiveness. Following this framework, the primary outcomes were Reach, Intervention Effectiveness, Adoption, Implementation, and Maintenance. These are assessed before, during, and after implementation. The research had a total duration of 14 months, with a follow-up measurement at 14 months. Data will be analyzed using multilevel modeling.

**Results:**

The study was funded in April 2018. Data collection occurred between November 2018 and January 2020. In total, 21 teams of 4 mental health institutions agreed to participate. Data analysis is ongoing and results are expected to be published in December 2020.

**Conclusions:**

This implementation research may provide important information about the implementation of psychosocial interventions in practice and may result in a program that is useful for Cognitive Adaptation Training, and possibly for psychosocial interventions in general.

**Trial Registration:**

The Netherlands Trial Register (NL7989); https://www.trialregister.nl/trial/7989.

**International Registered Report Identifier (IRRID):**

DERR1-10.2196/17412

## Introduction

Modern views on psychiatric treatment for people with severe mental illnesses are fundamentally different from the treatment views and practices decades ago. Whereas the former treatment for people with severe mental illnesses was predominantly provided in large institutions, a paradigm shift towards recovery-oriented treatment aimed at increasing participation in the community resulted in a considerable decrease in hospital-based care [1]. Yet, a small group of people, presenting treatment resistant positive symptoms, persistent negative symptoms, cognitive impairments, and functional impairments, still require high levels of support and ongoing treatment in inpatient facilities for a longer period of time [2].

Treatment and support in these facilities mostly consist of support in daily living activities by psychiatric nurses, pharmacotherapy, psychological therapy such as psychoeducation and cognitive behavioral therapy, and occupation- and work-related daytime activities. Often, the available evidence-based psychiatric rehabilitation interventions, such as Individual Placement and Support [3] and the Boston Psychiatric Rehabilitation Approach [4], are not feasible due to the cognitive and communicative impairments of the service users. A rehabilitation intervention that has been found to be effective in this population is Cognitive Adaptation Training [5]. Cognitive Adaptation Training aims to reduce functional problems caused by cognitive deficits through the use of compensatory strategies and environmental supports. The compensatory strategies and environmental supports are set up based upon an evaluation of the environmental context and functional skills, an assessment of cognitive strengths/weaknesses, and an assessment of how the cognitive problems are expressed in daily life. The effectiveness of Cognitive Adaptation Training has been investigated in a number of randomized controlled trials in various contexts (outpatients and inpatients) with consistent positive findings [6,7].

Although there is a convincing body of evidence describing the clinical value of Cognitive Adaptation Training in different settings and target groups (community care, residential facilities, first-episode psychosis) [5-13], implementation of the intervention into routine care has yet to be established. Literature on the implementation of evidence-based practices shows that this so called science-to-service gap is a widespread problem in both somatic and mental health care [14]; treatment guidelines are often not followed and as few as 8%-32% of the people with schizophrenia in an inpatient and outpatient setting are offered psychosocial interventions such as family psychoeducation or supported employment, despite the fact that it is part of their treatment plan [15]. Furthermore, research on the implementation of guideline recommendations in schizophrenia treatment showed that only 0%-7% of mental health care teams provide psychological or psychosocial evidence-based practices to more than 70% of the people in their caseload, even though these interventions were available to the teams [16]. After applying a series of structured activities aimed to implement evidence-based practices, these numbers increased to 10%-40% in the mental health care teams [16]. The reported factors that facilitated this implementation process were managerial support, a capable local team coordinator, and a motivated and skilled team of professionals. These studies show that dissemination of evidence-based practices alone does not lead to sustainable implementation, but when provided with the appropriate guidance, more sustainable implementation can be achieved.

To gain a better understanding of the processes involved in the implementation of Cognitive Adaptation Training, a posthoc process evaluation was conducted in a previous study [7] in order to clarify the providers’ needs and perspectives regarding the intervention and its implementation. The mainly qualitative findings indicated 3 important barriers to implementation: (1) knowledge and skills of the nursing staff to provide the intervention in the appropriate way, (2) organizational preconditions such as time and support, and (3) motivation of the nursing staff to provide the intervention to the service users. These findings were in line with the factors outlined in the COM-B model [17] that was designed to understand, explain, and influence behavioral change. The model assumes that causal and reciprocal relationships exist between 3 factors: capability, opportunity, and motivation and that these concepts influence and determine behavioral change. *Capability* is defined as the mental and physical ability of an individual to perform a certain activity, such as knowledge and skills. *Opportunity* is defined as all factors that are beyond an individuals’ sphere of influence yet enable or hinder the individual in performing certain behavior. *Motivation* is defined as all mental processes that bring about goal-directed behavior. It includes automatic and unconscious behavior, emotional reactions, and rational decision making that lead to certain behavior.

In this research, we propose and evaluate an implementation program that is based upon the results of our previous study’s process evaluation [7] and is theoretically grounded in the COM-B model. By putting this implementation program into practice, we aim to achieve a sustainable implementation of Cognitive Adaptation Training in routine mental health care. The primary aim of this research was to assess the effectiveness of the implementation program, which is referred to as *implementation effectiveness* throughout the article. The RE-AIM framework [18] is used to assess and report the implementation effectiveness and is defined by reach, effectiveness of Cognitive Adaptation Training, which is referred to as *intervention effectiveness* throughout the article, adoption, implementation (or fidelity), and maintenance. The secondary aim of this study was to evaluate the factors that impede or facilitate the implementation of Cognitive Adaptation Training.

## Methods

### Study Design

This study is a 2-arm multicenter cluster randomized controlled trial comparing the implementation program, consisting of tailored multifaceted implementation strategies to a preset program with single implementation strategies, using mixed methods. The total study duration was 14 months. The first 2 months are used to train the participating teams in Cognitive Adaptation Training. The assessments are administered at baseline (2 months; T0), 5 months (T1), and 8 months (T2). Long-term effects were assessed by a follow-up assessment at 14 months (T3).

### Setting

In total, 21 rehabilitation teams of 4 mental health institutions across The Netherlands were included. The size and compositions of the teams differed: the team sizes ranges from 4 to 28 team members, and the number of service users they help in their day-to-day needs ranges from 9 to 35. Most of the team members were psychiatric nurses with a degree at bachelor or below bachelor level. All teams provide long-term daily clinical care in an inpatient setting to adult people diagnosed with a severe mental illness according to DSM-IV or DSM-5 criteria, depending on the date of diagnosis. The majority of the service users receiving treatment were diagnosed with schizophrenia or related psychotic disorders; other diagnoses included severe depression, bipolar disorder, personality disorder, and autism. The teams were different from those that participated in the randomized controlled trial evaluating the effectiveness of Cognitive Adaptation Training [7] but were similar with regard to the provided treatment, support, and living conditions. The teams aim to provide a combination of treatments such as pharmacotherapy, psychological, psychosocial, and nonverbal therapies. Examples are psychoeducation, cognitive behavioral therapy, trauma therapy, creative therapy, and work projects such as landscaping, catering, woodworking, and production work.

### Participants

Participants in this study were members of the rehabilitation teams (including their managers) and service users. The members of the rehabilitation teams include nurses, social workers, peer support workers, and other professionals who provide day-to-day care to the service users. Treatment staff (psychiatrists, psychologists, and nurse practitioners) of the rehabilitation teams were excluded. The manager was defined as the person with managerial authority who monitors the functioning of the team members. Service users who receive outpatient treatment or those who were under the age of 18 were excluded from participation. No other inclusion or exclusion criteria were used.

### Recruitment and Allocation

 Two mental health institutions included in this study indicated that they were interested in Cognitive Adaptation Training before the start of the study. The managers of the rehabilitation teams of these mental health institutions and 2 other institutions were approached by the research group and were provided with information about the study. If they indicated that they were interested, an appointment was made to provide more in-depth information about the intervention, the research and the required investment in terms of time and effort. All managers of the participating mental health institutions were asked to sign a research statement. In this statement, the departments declared that the researchers and departments had sufficient expertise and facilities to conduct the research, that these facilities were available to the researchers, and that they would inform all people who were required to contribute to the research. In addition, a liability statement was included. The service users received both written and oral information about the research by the team members. All participants were asked to provide written consent to participation and were informed that their participation was voluntary and that withdrawal of consent was possible at any time without consequences.

To avoid contamination between the 2 treatment conditions, cluster randomization was applied at the team level. Teams were clustered if their members were situated in the same building or if they indicated that they interact with each other on a day-to-day basis. When these criteria did not apply, single teams were entered as a cluster. The teams were randomly allocated to either the experimental condition (multiple tailored implementation strategies) or control condition (single implementation strategies) by an independent staff member who blindly drew a ticket from a box containing a ticket for either condition.

### Cognitive Adaptation Training

The intervention to be implemented in this study was Cognitive Adaptation Training, a psychosocial intervention aimed at reducing functional problems caused by cognitive deficits by using compensatory strategies and environmental supports. Cognitive Adaptation Training was provided by the nurses and other team members who provide day-to-day care to the service users. Individual Cognitive Adaptation Training plans were set-up and tailored to the individual through gathering information regarding: (1) daily functioning (Environmental and Functional Assessment) [19], (2) cognitive functioning (Modified Wisconsin Card Sorting Test; Letter fluency test) [20,21], and (3) reflection of cognitive impairments in daily life (behavior type: apathy versus disinhibition (Frontal Systems Behavior Scale) [22]. Goals are determined through shared-decision making and based upon the daily functioning outcomes. Behavior type is used as a basis for designing compensational strategies: for example, for apathy, strategies involve cueing and prompting behavior, while for disinhibition, strategies are focused on removing irrelevant or distracting stimuli from the environment where the activity takes place. Information on cognitive functioning was used to tailor the environmental supports to the level of cognitive functioning of the individual (ie, global or step-by-step description).

### The Implementation Program

**Figure 1:**
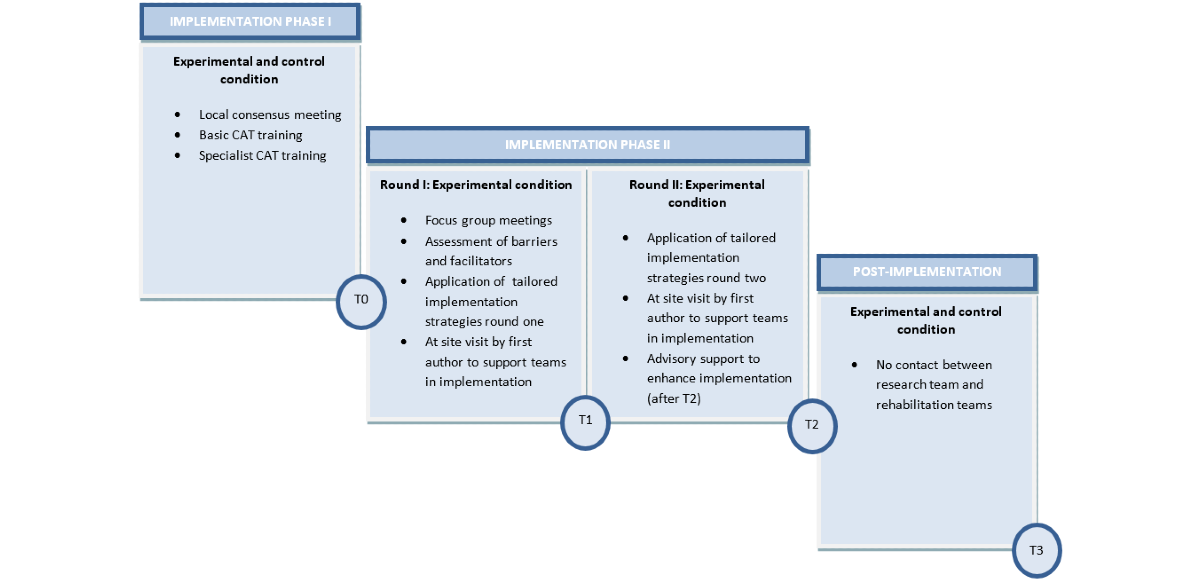
Implementation program. CAT: Cognitive Adaptation Training.

The implementation program was a 2-phase process with phase 1 being identical for all teams regardless of condition. This first phase included a local consensus meeting, a basic training in Cognitive Adaptation Training for all team members at site, and a specialist training in Cognitive Adaptation Training for a maximum of 2 team members per participating team. Phase 2 was offered to the teams in the experimental condition only and entailed the provision of multiple implementation strategies tailored to the needs and context of the individual teams. A graphical representation of the implementation program is presented in Figure 1.

### Implementation Phase 1: Experimental and Control Condition

#### Local Consensus Meetings

In all participating teams, a local consensus meeting was conducted by author MD to create a solid support base. In this meeting, information about Cognitive Adaptation Training, the implementation process, and the related research activities was provided to all team members. Moreover, this meeting was used to create commitment to the implementation study and for providers to decide whether to agree to participate.

#### Basic Cognitive Adaptation Training

Small educational group meetings were organized for all individual teams to educate them in the basic principles of Cognitive Adaptation Training. The basic training in Cognitive Adaptation Training was a single 90-minute meeting in which information was provided about the rationale behind Cognitive Adaptation Training and the steps involved to set up an individual Cognitive Adaptation Training plan. The educational meeting was provided on site by authors MD, LM, or a trained research assistant. It included an interactive component by training the team members in administering the Frontal Systems Behavior Scale, which is used for determining the compensational strategy.

#### Specialist Cognitive Adaptation Training

The qualitative results of the process evaluation as a part of the effectiveness study, which was conducted in a similar setting, showed that demoralization, indifference, and resistance of professionals to provide Cognitive Adaptation Training to the service users were important barriers to implementation. To overcome these barriers, local champions were recruited to promote the implementation process. The local champions were team members on site who were committed to the intervention and to performing the activities necessary to set up a Cognitive Adaptation Training plan. Two champions were recommended for each team and they were recruited on a voluntarily basis. They received 3-day specialist training provided by first author MD in which they gathered in-depth knowledge on Cognitive Adaptation Training–related constructs (ie, cognition, executive functioning, apathy, disinhibition), administering the interview and cognitive tests, on-the-spot training and supervision, designing a Cognitive Adaptation Training plan, and choosing appropriate environmental supports. At the end of the training, all champions received the materials and documents to provide Cognitive Adaptation Training to the service users.

### Implementation Phase 2: Experimental Condition

The second part of the implementation study was exclusively provided to the teams randomized to the experimental condition. The teams in the control condition received no further implementation support. An overview of all measurement instruments and assessment schedule is presented in Table 1.

**Table 1:** Schedule of assessments.

Level, Construct		Instrument	Baseline (T0; 2 months)	5 months (T1)	8 months (T2)	14 months (T3)
**Service user**						
	Demographical information		X	—	—	—
	Effectiveness	LSP^a^	X	—	—	X
		NOSCA^b^	X	—	—	X
**Mental health care staff**						
	Demographical information		X	—	—	—
	Capability	CAT Fidelity^c^	—	X^d^	X	X
	Opportunity	TCI^e^	X	—	—	X
		OCM^f^	X	—	—	X
	Motivation	EPBAS^g^	X	—	—	X
	Reach		—	—	X	X
	Adoption	MIDI^h^	—	X^d^	X	X
	Implementation	CAT Fidelity	—	X^d^	X	X
	Maintenance		—	—	—	X
**Manager**						
	Recovery-oriented care	ROPI^i^	X	—	—	X

^a^LSP: Life Skills Profile.

^b^NOSCA: Nurses’ Observation Scale of Cognitive Abilities.

^c^CAT Fidelity: Cognitive Adaptation Training Fidelity Scale.

^d^Assessed for the teams in the experimental condition only.

^e^TCI: Team Climate Inventory.

^f^OCM: Organizational Climate Measure.

^g^EPBAS: Evidence-Based Practice Attitude Scale.

^h^MIDI: Measurement Instrument for Determinants of Innovation.

^i^ROPI: Recovery Oriented Practices Index.

### Experimental Condition: Round 1

#### Focus Group Meetings

Two focus group meetings were organized by first author MD and a trained research assistant in each mental health institution for the teams in the experimental condition. The purpose of these focus group meetings was to collect qualitative data on the status quo regarding the quality of care that is provided to the service users and to gather additional information on barriers and facilitators to implement Cognitive Adaptation Training in the context of the COM-B constructs (capability, opportunity, motivation, and behavior change). For example, information was gathered on the perspective of the focus group participants regarding the extent to which the teams provide recovery-oriented care, how they support the service users in pursuing their own personal goals, how this is integrated in their day-to-day care, and what can be improved to optimize this process. The interview guide was developed by author MD and was modified in consultation with authors JW, MP, SC, and LM.

In a previous experience with combined focus groups (mental health professionals and service users), we noticed that service users could not keep up with the speed of the discussion. Therefore, we deliberately choose to split the focus groups. The first focus group included service users only, and the second focus group was organized for family members, caregivers, and mental health professionals. We aimed to include a minimum of 5 and maximum of 8 participants in each focus group.

#### Assessment of Barriers and Facilitators Based on COM-B

To assess implementation barriers and facilitators in each individual team, all team members who were involved in the day-to-day care of the service users received a set of questionnaires measuring different components of the COM-B model. The questionnaires were distributed by an email that included a personal link. The link to the questionnaires could only be used once after completion to avoid duplicate entries from the same individual. Responses were recorded automatically. Informed consent was obtained online. Online data collection was performed according to the guideline for online surveys, where applicable [23]. The measurement instruments used in this assessment are described below.

#### Team and Organizational Climate

The *opportunity* of the local champions to implement Cognitive Adaptation Training in their teams was measured on 2 levels: team level and organizational level. The Team Climate Inventory [24] was used to measure team climate. It is a self-report questionnaire that measures 38 items on a 5-point scale (total score range: 38-190). The Team Climate Inventory consists of 4 scales, subdivided into 13 subscales: (1) participative safety (information sharing, safety, influence, and interaction frequency), (2) support for innovation (articulated support, enacted support), (3) vision (clarity, perceived value, sharedness, attainability), and (4) task orientation (excellence, appraisal, and ideation). The translated Dutch version has been validated and shows good psychometric properties [25].

The Organizational Climate Measure [26] is a self-report questionnaire measuring 82 items on a 4-point scale (total score range: 81-324). The scale contains 17 subscales, divided into 4 quadrants: (1) human relations (autonomy, integration, involvement, supervisory support, training, welfare), (2) internal process (formalization, tradition), (3) open systems (innovation and flexibility, outward focus, reflexivity), and (4) rational goals (clarity of organizational goals, efficiency, effort, performance feedback, pressure to produce, quality). The Organizational Climate Measure was translated to Dutch. The English version of the Organizational Climate Measure has good psychometric properties [26].

#### Motivation and Attitudes

To measure the *motivation and attitudes* of the team members to adopt evidence-based practices in the teams, the Evidence-Based Practice Attitude Scale [27] was used. The Evidence-Based Practice Attitude Scale is a self-report questionnaire consisting of 15 items measured on a 5-point Likert scale (total score range: 0-60) and consists of 4 subscales: (1) appeal, (2) requirements, (3) openness, and (4) divergence. The translated Dutch version of the Evidence-Based Practice Attitude Scale has good psychometric properties [28].

#### Implementation Strategies

The implementation efforts for the teams in the experimental condition included fixed and flexible implementation strategies based upon the literature [29]. The fixed implementation strategies were (1) a feedback meeting to discuss the results of the questionnaires measuring potential implementation barriers and facilitators, (2) collaboratively deciding on the implementation strategies in the teams, (3) on-site support by the first author in establishing the strategies, (4) a process evaluation at 3 months to measure the effect of the implementation strategies, (5) collaboratively deciding whether to change the implemented strategies, (6) a second on-site support visit by the first author to support the implementation strategies, and (7) an advisory report set up together with the local champions that describes potential implementation strategies. The flexible implementation strategies depended on the results of the feedback meeting and the process evaluation for each individual team. This part was context dependent and had to be tailored to the needs and resources of the individual teams. If there was no budget or support base to implement a certain strategy, then we collaboratively had to find an alternative. Therefore, the flexible part could not be determined in advance but had to be decided during the course of the implementation period. For the implementation a process, progress, selection, and adaptation of strategies logbook was kept throughout the study period. The fixed and examples of flexible implementation strategies are described below.

In each individual team in the experimental condition, a feedback meeting was organized with the team members to discuss the results of the questionnaires measuring implementation barriers and facilitators. Implementation strategies that match the barriers and facilitators for each individual rehabilitation team were reviewed and were determined in consensus. For example, when team members indicated that there was limited time to implement Cognitive Adaptation Training in their daily working routine, we might discuss options to deimplement routines or tasks that have little or no beneficial effect. Or we might discuss options to alter Cognitive Adaptation Training in a way that it did not affect fidelity but would make it a group effort so that it was less time consuming for the local champions. Next, the tailored implementation strategies collaboratively selected in the feedback meeting were applied in practice. The researcher supported the individual teams in the implementation activities by visiting them once during the first 3 months.

#### Process Evaluation

After 3 months, a process evaluation was administered to assess the implementation progress and to re-evaluate the implementation strategies. If the results indicated that implementation barriers or facilitators had shifted or if the tailored implementation strategies did not show the desired results, new strategies were developed and applied. The process evaluation consisted of 2 interviews with each individual local champion by the first author or a research assistant: the CAT Fidelity Scale and the Measurement Instrument for Determinants of Innovation (MIDI) [30].

The *capability* of the local champions to provide Cognitive Adaptation Training as intended by the program developers is measured by the CAT Fidelity Scale. The CAT Fidelity Scale was developed in collaboration with research groups from the United States, Canada, Australia, Sweden and Finland (publication in preparation). Following the Delphi method [31], a multidisciplinary panel of experts was asked to reach consensus on the items. This resulted in a 6-point scale that comprises 44 items (total score range: 0-220) and measures various aspects related to Cognitive Adaptation Training: characteristics of the Cognitive Adaptation Training-specialist, administration and organizational requirements. Two raters score the CAT Fidelity Scale independently. If there is a disagreement among the raters on certain items, these items are discussed to reach consensus. A preliminary evaluation showed moderate interrater reliability of the scale (κ=0.51).

To gain a better understanding of the factors that influenced the implementation of Cognitive Adaptation Training, the MIDI [30] was used. The MIDI is a semistructured interview measuring 27 determinants on a 5-point scale. The determinants are subdivided into 4 domains: determinants associated to the innovation, the adopting person, the organization, and the sociopolitical context (ie, whether the activities and procedures of Cognitive Adaptation Training are in accordance with legislation and regulations). The MIDI was developed based upon a systematic review and a Delphi study among implementation experts, thereby ensuring content validity. Although psychometric properties have not yet been established, other studies in mental health administering the MIDI among health care professionals showed an internal consistency (Cronbach α) between .61-.93 [32,33].

### Experimental Condition: Round 2

The first author visited the individual teams of the experimental condition a second time after the process evaluation to provide support to the teams in the implementation activities. If new implementation strategies needed to be applied, this was discussed with the local champions. A second process evaluation was administered 6 months after the start of the implementation. Following this second process evaluation, an advisory report was set up by the first author in collaboration with the local champions in which suggestions for implementation strategies were described. The purpose of the advisory report was to provide suggestions to the local champions and the rest of the team that would help to better implement Cognitive Adaptation Training in their daily working routine. By providing them with advice, rather than physically guiding them as in the first 2 phases, we aimed to allow the teams to become more autonomous and inventive in setting up implementation strategies in the face of future barriers. Furthermore, as the advisory report was drafted based upon the results of the process evaluation, the advisory report in itself was an implementation effort as we informed the teams on the progress of implementation. The researchers and the teams in the implementation condition were not in contact with each other in between the advisory report and the follow-up assessment (T3) to discuss implementation problems or implementation activities.

### Outcomes and Measures

The outcome evaluation of the implementation program was based upon the RE-AIM framework [18]. This framework describes implementation success as a combination of reach, intervention effectiveness, adoption, implementation, and maintenance. In addition to the instruments that were selected based upon the RE-AIM framework, the questionnaires described in Assessment of Barriers and Facilitators and Process Evaluation sections were also used as outcome measures.

*Reach* was measured by the proportion of service users who received Cognitive Adaptation Training in each team and by comparing these numbers between the 2 treatment arms. To detect possible selection bias, the representativeness of service users who received Cognitive Adaptation Training in the intervention condition was determined by comparing their demographic variables to those of the service users in the entire study population.

*Intervention effectiveness* refers to the clinical improvement of the service users as a result of Cognitive Adaptation Training. The effectiveness of Cognitive Adaptation Training was determined by the improvements of the service users on daily functioning and cognitive functioning measured by 2 observational questionnaires: Life Skills Profile [34] and the Nurses’ Observation Scale of Cognitive [35]. The Life Skills Profile consists of 39 questions on a 4-point scale (total score range: 39-156) and measures various aspects related to daily life activities: self-care; nonturbulence; social contact; communication; and responsibility. Results on the Life Skills Profile from the previous research [7] evaluating the effectiveness of Cognitive Adaptation Training revealed a significant effect for people receiving Cognitive Adaptation Training in addition to treatment as usual compared to treatment as usual only. The Nurses’ Observation Scale of Cognitive Abilities [35] measures cognitive functioning and includes 39 items (total score range: 0-121) subdivided into 8 cognitive domains (subscales): attention, perception, memory, orientation, higher cognitive domains, thoughts, language, and praxis. The Nurses’ Observation Scale of Cognitive Abilities is scored on a 4-point scale. Both the Life Skills Profile and Nurses’ Observation Scale of Cognitive Abilities have good psychometric properties [36,37].

*Adoption* was defined as the representativeness of participating sites and intervention agents that adopted the intervention. It is measured at the system level by comparing the determinants of the MIDI across the 2 implementation conditions.

*Implementation* refers to the Cognitive Adaptation Training specialists’ fidelity to the elements of Cognitive Adaptation Training as described in the Cognitive Adaptation Training protocol. The level of implementation was measured by the CAT Fidelity Scale.

*Maintenance*, or sustainability of the intervention was evaluated at follow-up, which was 6 months after the final contact with the implementation researchers. Maintenance was measured by comparing the differences between postmeasurement and follow-up of the abovementioned constructs (reach, intervention effectiveness, adoption, and implementation) between the 2 conditions.

Demographic information was obtained from both service users (birth year, gender, nationality, level of education, main diagnosis, age of onset) and the team members (date of birth, gender, nationality, level of education, years working experience, years working with the target group population, years working in current team) and was completed at baseline assessment.

### Recovery-Oriented Practice

To measure the extent to which the teams provided recovery-oriented care in general, the translated Dutch version of the Recovery Oriented Practices Index [38] was used. It consists of 26 items on a 5-point scale (total score range: 0-130), measuring 8 domains of recovery-oriented care. The domains include meeting basic needs; comprehensive services; network supports and community integration; service user involvement and participation; strengths-based approach; customization and choice; self-determination; recovery focus. The Recovery Oriented Practices Index was administered and scored by one of the researchers through an interview with the management of the rehabilitation teams. The construct validity of the Recovery Oriented Practices Index has been reported as good [39].

### Sample Size

Sample size was estimated for both intervention effectiveness of Cognitive Adaptation Training (based upon the Life Skills Profile) as well as implementation effectiveness (based upon the outcome variable Reach). To ensure enough power for both purposes, we used the highest estimated sample size. For intervention effectiveness, with a mean difference of 6 points on the Life Skills Profile (considered clinically relevant according expert opinions) and a standard deviation of 10.5 (based on the effectiveness trial of Cognitive Adaptation Training in residential settings [7], 78 service users needed to be included to detect significant improvements on the Life Skills Profile with a power of .8 and a significance level of .05. Accounting for a conservative attrition rate of 20%, a minimum of 98 service-users needed to be included in the study. For implementation effectiveness, based upon previous implementation research in the Netherlands [16], the expected proportion of service users reached in the experimental condition was set at 30%, compared to 5% in the control condition. To be able to detect a difference between conditions in proportion of service users reached with Cognitive Adaptation Training (with α=.05 and a power of .8), both groups (control and experimental) required a caseload of at least 34 service users each.

### Statistical Analyses

To measure demographic and baseline differences between the 2 groups, chi-square tests will be performed for categorical variables, and 2-tailed independent sample *t* tests will be performed for continuous variables using SPSS software (version 24.0; IBM Corp). To assess the implementation effectiveness over time (outcome evaluation), mean differences in outcomes between the 2 arms on the dimensions of the RE-AIM framework were assessed using multilevel modelling [40]. A 3-level model will be built with team (level 3), subjects (level 2), and assessment (level 1) entered as levels. Significance of the fixed regression effects will be tested using the 1-tailed independent sample *t* test (=.05). The content of the focus group meetings and process evaluations will be transcribed verbatim and analyzed using a combined approach of inductive and deductive thematic analysis in ATLAS.ti (version 8.0; ATLAS.ti Scientific Software Development GmbH).

### Ethics and Data Privacy

The Medical Ethics Review Committee of the University Medical Center Groningen in the Netherlands (file number: M17.220439) concluded that the study did not fall within the scope of the Medical Research Involving Human Subjects Act and waived the requirement for ethical approval. The study was conducted in compliance with local and international ethical standards and the Declaration of Helsinki [41]. Additional documents related to the ethical protocol can be requested from the corresponding author. The final and complete data set will only be accessible to members of the research team. Procedures regarding data management are in compliance with the Research Data Policy of the University of Groningen.

## Results

The study was funded in April 2018 by a grant of the Foundation for Support (*Stichting tot Steun* VCVGZ; grant number: 247). The study was retrospectively registered at the Netherlands Trial Register in September 2019 (NL7989). Data collection occurred between November 2018 and January 2020. In total, 21 teams of 4 mental health institutions agreed to participation. Data analysis is ongoing, and the results are expected to be published in December 2020.

## Discussion

The implementation of evidence-based practices in mental health care has received increased attention over the last few years. For example, funding agencies now request a detailed description of plans for implementation and dissemination activities to translate the research findings into clinical practice. Yet to date, evidence-based practices are rarely available for people with severe mental illness in various treatment settings [15,16]. One such evidence-based practice is Cognitive Adaptation Training, which aims to enhance independent daily functioning in people with severe mental illness. To improve implementation success of Cognitive Adaptation Training in clinical practice, a novel implementation program was developed that uses a systematic approach while considering context dependent factors. In this study, the effectiveness of this implementation program will be assessed. An effective implementation program will enhance the implementation success of Cognitive Adaptation Training on a broad scale and hence facilitate recovery in a group of people that is relatively underrepresented in the scientific literature.

An important strength of this study is its comprehensive design. The recruitment of departments in multiple mental health care organizations across The Netherlands increases the representativeness and the generalizability of the results. An additional strength in the study design is the use of mixed methods. This design enables us to explore and obtain an in-depth understanding of the processes involved during implementation, which will help to interpret the results at the end of the study. Furthermore, using both quantitative and qualitative measures to identify tailored strategies for implementation provides a more comprehensive perspective than either approach alone. By gathering this information on multiple levels of care (service user level, provider level, manager level), the Cognitive Adaptation Training program ensures a holistic approach in which all stakeholders' needs and perspectives are considered. Another strength of this study is that the implementation program is tailored to the needs of the individual teams. By matching the implementation strategies to the team and organizational context and collectively deciding which implementation strategies are acted upon and which strategies need reconsideration, rather than adopting a one-size-fits-all approach, we aim to increase the implementation success.

We do not include cognitive performance in service users as an outcome measure. Given that we demonstrated cognitive improvements related to Cognitive Adaptation Training in another recent multicenter randomized controlled trial [7], one might consider this a minor limitation in this study. However, as it is not the goal of Cognitive Adaptation Training to improve cognition, but rather to bypass cognitive impairments, cognition is not included as a primary outcome measure in the aforementioned multicenter randomized controlled trial. Thus, since we focus upon implementation effectiveness in this study and to relieve the burden for service users, we felt it would be appropriate to use an observational questionnaire measuring cognition as a valid substitute. Although we did not find improvements on all cognitive domains in the randomized controlled trial evaluating the effectiveness of Cognitive Adaptation Training, the Nurses’ Observation Scale of Cognitive Abilities predominantly measures domains related to frontal lobe functioning (thought processing, orientation, memory, attention, and consciousness). Since our previous study [13] on Cognitive Adaptation Training showed significant effects in visual attention and executive functioning, we considered it to be justified to include a measure that covers these domains and other cognitive domains related to frontal lobe functioning.

A second limitation to this study is the potential threat to nonresponse and selection bias. Even though the study was carefully designed to minimize the time-consuming burden of filling out questionnaires for both the health care professionals and service users, we recognize that it requires an extra effort in their already demanding day-to-day jobs. As a result, some health care professionals may not respond to the online questionnaires, causing nonresponse and selection bias. Also, as some of the included institutions showed interest in participation before the start of the study, this may influence the results and thus limit the generalizability. This should be taken into consideration while analyzing and reporting the final results.

The implementation program presented in this study can help to bridge the science-to-service gap in mental health care and may provide important information regarding facilitators and barriers to implementation for other mental health researchers and implementation scientists. Moreover, when proven effective, this implementation program may also be effective for the implementation of other psychosocial interventions or innovations in long-term psychiatric care.

## Appendix

Multimedia Appendix 1 Peer Review Report.

## Abbreviations

CAT Fidelity Scale: Cognitive Adaptation Training Fidelity Scale
COM-B: Capability Opportunity Motivation–Behavior change
DSM-IV: Diagnostic and Statistical Manual of Mental Disorders (Fourth Edition)
DSM-5: Diagnostic and Statistical Manual of Mental Disorders (Fifth Edition)
MIDI: Measurement Instrument for Determinants of Innovation
RE-AIM: Reach Effectiveness Adoption Implementation Maintenance
