# Supplementary material for: Evaluation of an Adaptive Implementation Program for Cognitive Adaptation Training for People With Severe Mental Illness: Protocol for a Randomized Controlled Trial
Source: JMIR Res Protoc. 2020 Aug 24;9(8):e17412. doi: 10.2196/17412 (PMC7477665; doi:10.2196/17412)
Supplement: Multimedia Appendix 1 [file resprot_v9i8e17412_app1.pdf]

Reactie vragen VCVGZ – CAT implementatieonderzoek

1. Effectiviteit van de implementatiestrategie.

De effectiviteit van de implementatieaanpak lijkt goed. Het gebruik van een multifaceted, planmatige aanpak met 'tailored' strategieën welke op maat worden gemaakt voor de lokale context m.b.v. het COM-B model lijkt passend en effectief. Een nadeel van een dergelijke 'tailored' aanpak is dat je vooraf niet exact weet welke strategieën toegepast gaan worden, maar het geeft vertrouwen dat er gebruik wordt gemaakt van een voorselectie van strategieën passend bij de COM-B concepten, literatuuronderzoek voor effectiviteit van strategieën, focusgroepen voor feedback op de selectie van strategieën en evaluatie op de toepassing van de strategieën.

2. Het feit dat er geen vergelijkende studie wordt gedaan om een assessment op de langere termijn te maken.

In het voorstel wordt de 'vernieuwde implementatiestrategie' in het voorliggende onderzoek vergeleken met de implementatiestrategie uit de eerdere multi-site RCT (welke kan worden opgevat als 'concurrerende' of 'gebruikelijke' of implementatiestrategie). Hiermee kan de primaire onderzoeksvraag (effectiviteit van de implementatiestrategie) voor de uitkomstmaat 'bereik' mijn inziens zeker goed beantwoord worden. Het is mij niet helemaal duidelijk waarom voor de uitkomstmaten 'modeltrouw' en 'continuïteit' geen vergelijking met de eerdere RCT wordt gemaakt, mogelijk omdat deze maten destijds niet (op eenzelfde) manier zijn gemeten. Het berekenen van het verschil in bereik en modelgetrouwheid tussen de verschillende evaluatiemomenten binnen het huidige onderzoek (waarbij het laatste evaluatiemoment plaatsvindt als er geen betrokkenheid van het onderzoeksteam meer is) lijkt me dan een goed alternatief.

3. Daarnaast vraagt het bestuur zich af of de huidige onderzoeksopzet niet te veel subonderzoeken bevat, die geen verband houden met de implementatie.

Het aantal sub-onderzoeken (de doelgroep-analyse m.b.v. een IPD-MA, de testbetrouwbaarheid analyse van de CAT fidelity schaal, naast het implementatieonderzoek met de primaire, secundaire en procesmaten) lijkt inderdaad vrij groot, vooral voor het huidig aangevraagde budget, de inzetbaarheid van de junior onderzoeker en de doorlooptijd van het onderzoek. Onze ervaring is dat alleen al het uitvoeren van een IPD-MA een tijdrovende activiteit is (onder andere i.v.m. het opvragen van oorspronkelijke data, en de data-cleaning vanwege verschillende meetmethoden in de verschillende trials) waarvoor wij al snel op een inzet van 60 dagen komen bij 'dubbel reviewen' en 40 dagen bij 'enkel reviewen'. De benodigde inzet voor de tetsbetrouwbaarheid analyse kan ik moeilijk inschatten, maar het totale pakket lijkt inderdaad vrij ambitieus.
